# Robotic training in transplant surgery fellowship: shaping the next generation of transplant surgeons

**DOI:** 10.1007/s11701-026-03215-w

**Published:** 2026-02-19

**Authors:** Caleb Harsin, Amen Kiani, Neeta Vachharajani, Angela Hill, Meranda Scherer, Jesse T. Davidson, Jason R. Wellen, Jessica Lindemann, Majella B. Doyle, William C. Chapman, Adeel S. Khan

**Affiliations:** 1 Section of Abdominal Transplant, Department of General Surgery, Washington University in St. Louis, St. Louis, MO 63110 USA; 2 4921 Parkview Place #12B, St. Louis, MO 63110 USA

**Keywords:** Robotics, Transplant fellowship, Surgical training, Curriculum, Education, Surgical simulation

## Abstract

The indications for robotic surgery in transplant and HPB procedures are rapidly expanding. However, there is growing concern about the adequacy of robotic training during fellowship in preparing trainees for the evolving demands of modern transplant surgery. This is a single center, retrospective review of the robotic experience of 9-abdominal transplant fellows (ASTS) over a 7-year period (2017–2024) at a high-volume center. A 10-question survey was administered to elicit fellow perspective on their training. The 9 fellows participated in 692 of the total 860 robotic cases (80%) performed on the transplant service during the study period. Case breakdown was 337 (49%) transplant and 355 (51%) HPB cases. Fellows performed 91% of the operations on the console and 9% as bedside assistant. All fellows believed that robotic fellowship training will improve a fellow’s job prospects, and 87% felt that indications for robotic approaches in transplant surgery will broaden significantly. Incorporating formal robotic training in transplant surgery fellowships enhances the fellowship experience by better preparing fellows for the challenges of modern transplant surgery.

**Supplementary Information:**

The online version contains supplementary material available at 10.1007/s11701-026-03215-w.

## Introduction

Abdominal transplant surgery has undergone a transformative change in recent years. Technological and biomedical advancements—ranging from normothermic and hypothermic perfusion to xenotransplantation, transplant oncology, and robotic surgery—are reshaping the field and making transplantation safer and more accessible than ever before [1, 2].

The indications for robotic surgery in both transplant and hepatopancreatobiliary (HPB) surgery have steadily expanded over the past decade [1–3]. Procedures such as living donor nephrectomy, donor hepatectomy, and kidney transplantation are now routinely performed robotically at many centers, with outcomes that are comparable to, and in some cases exceed, those achieved with traditional open and/or laparoscopic techniques [4–7]. More recently, early reports have demonstrated the feasibility and safety of robotic liver transplantation in select patients [8, 9].

While the role of robotics in transplant surgery is poised to grow, how to train the next generation of surgeons in these techniques remains unclear. General surgery residents are receiving more and more training in robotic surgery during residency; as incoming transplant and HPB fellows, they not only recognize the value of robotic skills in their future practice but also view such training as both professionally essential and strategically advantageous in a competitive job market [10]. The expectations of prospective fellows, coupled with the growing experience of transplant surgeons using robotic platforms, has prompted many programs to explore the integration of robotics into fellowship training [7, 10, 11]. However, implementing robotic training during transplant fellowship has been challenging. Many transplant surgeons remain early in their own robotic learning curves and are not yet comfortable in giving up controls to the fellows for many cases. Moreover, there is wide variability in scope of clinical practices in transplant centers which makes it difficult to standardize training across programs, especially in the absence of guidelines from transplant associations regarding robotic training during fellowship.

This paper presents a seven-year experience in training transplant fellows in robotic surgery at a high-volume tertiary care transplant center. While the specific approaches and trainee experiences described may reflect the unique structure and resources of a single institution, the strategies outlined offer insights into how robotic training can be effectively integrated into abdominal transplant fellowship programs.

## Methods

This is a single center, retrospective review of a prospectively maintained database of all robotic cases performed on the transplant surgery service since the inception of the robotic transplant program in 2017. The primary focus of interest was the evolution of robotic surgery experience for transplant fellows enrolled in the 2-year American Society of Transplant Surgery (ASTS) certified abdominal transplant fellowship over the last 7 years (July 1st, 2017, to August 31st, 2024). A total of 21 fellows (9 ASTS abdominal transplant fellows and 12 non-ASTS fellows [6 HPB surgery fellows from Washington University HPB Surgery program and 6 visiting surgical oncology fellows from Memorial Sloan Kettering Cancer Center [MSKCC], New York City, NY]) spent time on the robotic transplant service during the study period. The 12 non-ASTS fellows rotated on the transplant surgery service for 1 month each, and since they were not primarily part of the transplant fellowship, their cases were not included in the analysis. Robotic cases that were not covered by any fellow were also excluded from this study.

### Robotic curriculum

Details of the robotic curriculum have been described previously [1, 5, 11] and include.

1. Basic training: Educational videos and hands-on training for teaching positioning, docking, undocking, instrument exchange and troubleshooting, etc.
2. Training lab access for simulation exercises (camera and instrument control, efficiency of movement, suturing, etc.), inanimate drills, porcine and cadaver labs.
3. Robotic case video library access for step-by-step review of common transplant and HPB operations.
4. Operation specific training [11, 12].
  - Bio-Tissue Training models: These models have high haptic realism and provide realistic training in tissue handling and robotic anastomoses (LifeLike Biotissue Inc. [London, Ontario, Canada]) [14–16]. Models used for training are visualized in Online Resource 1 and include:
    i. Hepaticojejunostomy.
    ii. Pancreaticojejunostomy.
    iii. Gastrojejunostomy.
    iv. End-to-end and end-to-side vascular anastomosis model.
  - Kidney-Transplant Models (Online Resource 2).
  - Porcine labs (kidney transplant, hepatic resection).
  - Cadaver labs: (kidney transplant, liver transplant, hepatectomy, pancreatectomy)
  - Perfused cadavers: These are fresh cadavers with pressurized arterial and venous systems and provide a realistic training model for living donor liver resections, living donor nephrectomy and kidney transplant.
5. Intraoperative training pathways:
  - Intraoperative assessment and feedback on pre-specified portions of operations using standardized scoring system (RO-SCORE) [11, 12].
  - Video review feedback for index operations.
  - Advanced Robotic Kidney Transplant Immersion Camp (ARKTIC): A graduated program for training fellows in robotic kidney transplants with the objective of having the fellows perform the operation independently (100% autonomy) by the end of ARKTIC.

Robotic cases performed by fellows were reviewed by academic year as well as by individual fellows. Cases were broadly categorized as either (1) Transplant or (2) HPB. Transplant cases were further sub-categorized into 5 groups: living donor nephrectomy, kidney transplant, living donor hepatectomy, liver transplant and others. The ‘other’ group included any robotic case performed on a transplant patient that did not meet the criteria for inclusion in the other 4 groups (e.g., transplant ureter stricture revision, transplant nephrectomy, or hepatic resection after liver transplant). HPB cases were sub-categorized into 5 groups as well: hepatic, pancreatic, biliary, foregut (gastric), or others (any robotic case in a non-transplant patient that was not related to liver, pancreas, biliary system, or stomach). Data collected also included the role of the participating fellow (console surgeon or bedside assistant), year of fellowship at time of case (first-year or second-year trainee), case complexity (low, medium, or high as categorized by the WashU Robotic Complexity Score [WURC] [13]), and whether the case was aided by a dedicated robotic first assist (RFA).

The 9 fellows were assigned values 1–9 in chronological order according to the year of completion of the 2-year fellowship. Fellow 1: 2017–2019, Fellow 2: 2018–2020, Fellow 3: 2019–2021, Fellow 4: 2020–2022, Fellow 5: 2021–2023, Fellow 6: 2021–2023, Fellow 7: 2022–2024, Fellow 8: 2023–2025, Fellow 9: 2023–2025. Individual years referenced throughout the paper refer to academic years (July 1 or August 1 to June 30 or July 31) and not calendar years. For example, 2017 refers to the academic year spanning July 1st, 2017, to June 30th, 2018. Seven fellows in the cohort completed the 2-years ASTS fellowship by the end of the study period while 2 fellows had completed only 1 year (fellow 8 and fellow 9). The expected 2-year robotic case volumes of the final 2 fellows were estimated by doubling the number of robotic cases they had participated in by the end of the first year of training (July 31, 2024). This was done to simplify calculations regarding the average number of cases completed per fellow during the fellowship; any calculations that include the projected case volumes are marked with an * (asterisk) symbol. This extrapolated number likely underestimates the final case number for these two fellows as the number of robotic cases has increased over each of the study years.

The 9-participating fellows completed a 10-point questionnaire regarding their views on robotic training in the transplant fellowship in general and the robotic experience during their respective fellowships (Online Resource 3).

## Results

During the study period, a total of 860 robotic cases were performed on the transplant surgery service, of which 692 (80.4%) were covered by the 9 ASTS-certified transplant fellows and make up the primary study cohort. An overview of the cases that made up the study cohort along with the details of fellow participation in these cases is included in Tables 1 and 2. Of the 692 robotic cases, 49% (*n* = 337) were transplant-related, and 51% (*n* = 355) were HPB. Within the transplant cohort, living donor nephrectomy made up the majority (75%) of cases, followed by kidney transplant (17%) and liver transplant (1%). There were no living donor hepatectomies with fellow participation during the study period. The HPB cohort included 38% biliary, 25% liver, 17% pancreas, and 8% gastric cases. The majority of fellow-performed cases were high complexity (68%), with moderate and low complexity cases each contributing 16% of the cohort. A dedicated bedside first assist was present for 669 (96%) cases. The fellows participated as console surgeons in 91% (633) cases and as bedside assistant for the remaining 9% (59) cases. Table 2 provides details of the individual robotic experience for the 9 fellows. On average, fellows performed 96 robotic cases during their two-year fellowship (45 transplant and 51 HPB cases). The breakdown for transplant cases included an average of 33 living donor nephrectomies, 7 kidney transplants, and a small number of liver transplant cases. The HPB sub-groups included an average of 13 hepatectomies, 10 pancreatectomies, 19 biliary, and 5 gastric cases.

**Table 1:** Overview of the robotic experience of the 9 ASTS abdominal transplant surgery fellows during the study period

	*N* (%)
Total robotic cases during study periodRobotic cases with fellow participation	860692 (80.4%)
**Case overview of robotic cases with fellow participation (** ***N*** ** = 692)**	**337 (49%)**
**Transplant**	
Living donor nephrectomy Kidney transplant Others **HPB** Liver Pancreas Biliary Gastric Other HPB related	232(75%) 57 (17%) 28 (8%) **355 (51%)** 87 (25%) 61 (17%) 136 (38%) 30 (8%) 41 (12%)
**Average number of robotic cases per fellow during 2-year fellowship** Living donor nephrectomy Kidney transplant Liver (HPB) Pancreas (HPB) Biliary (HPB)	**96.1** 33.3 7.4 12.8 9.8 19.4
Role of participating fellow	
Console surgeon Bedside assistant	633 (91%) 59 (9%)
Dedicated bedside assistant present	669 (96%)
Case complexity	
High Medium Low	473 (68%) 111 (16%) 108 (16%)

Average number of cases per fellow are also provided

*Fellowship robotic case numbers for the two fellows expected to graduate in 2025 are projected values based on case numbers at the end of first year of fellowship (Jul 31, 2024)

**Table 2:**
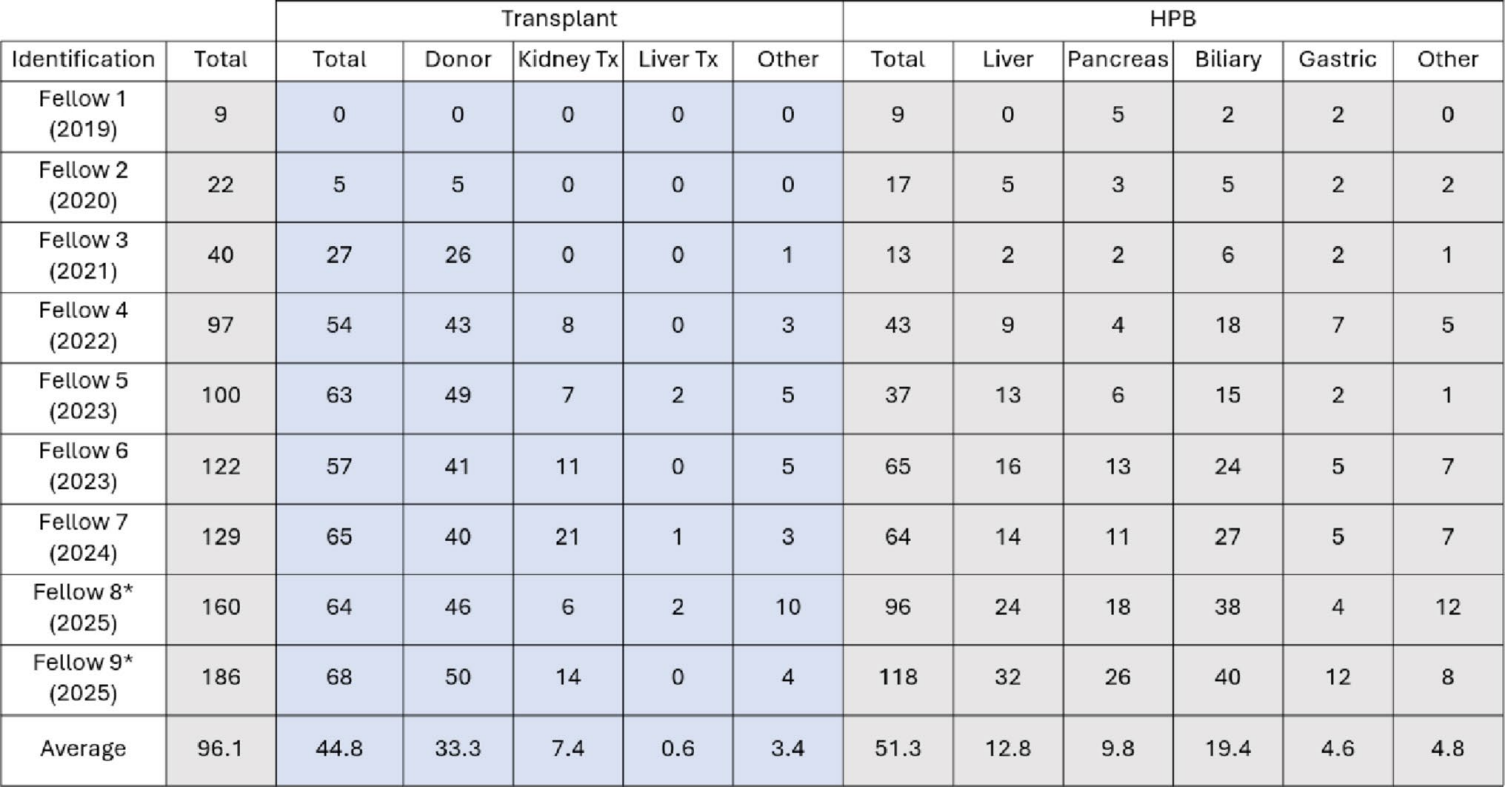
Details of the individual robotic case experience of the 9-fellows during the study period

*Robotic case numbers for fellows 8 and 9 have been extrapolated based on numbers at the end of first year of fellowship

**Fig. 1:**
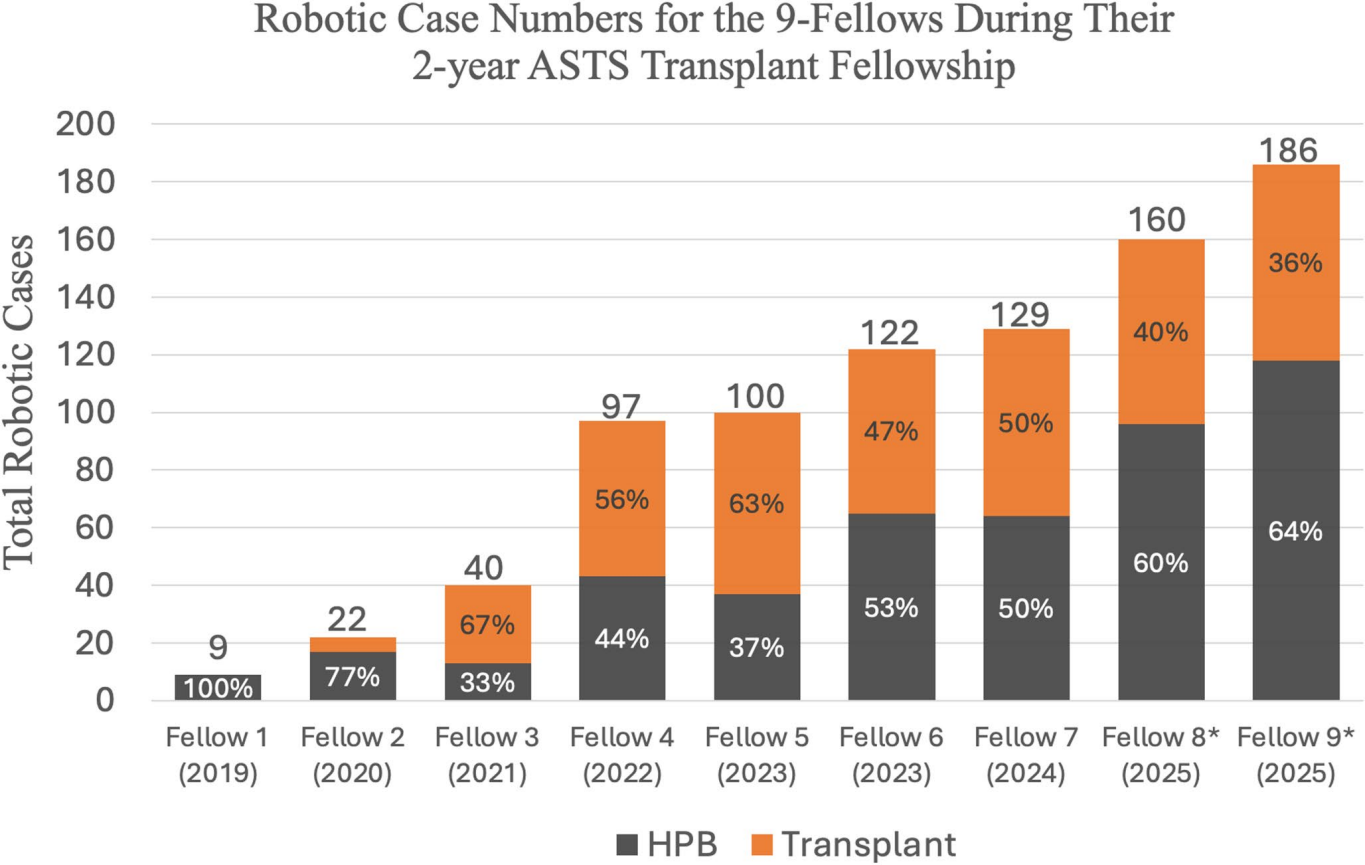
Graph representing robotic cases performed by each fellow during their two-year fellowship period. Fellows are arranged in chronological order with year of fellowship completion

Figure 1 gives a chronological illustration of how the individual robotic experience of the 9 fellows evolved over the course of the 7-year study period. Each subsequent fellow performed a higher case volume than their predecessor. The first two fellows in the study period performed 9 and 22 robotic cases respectively, almost all of which were HPB cases. The most recent fellows are projected to complete 160 and 186 robotic cases respectively by the end of their fellowship in July of 2025.

**Fig. 2:**
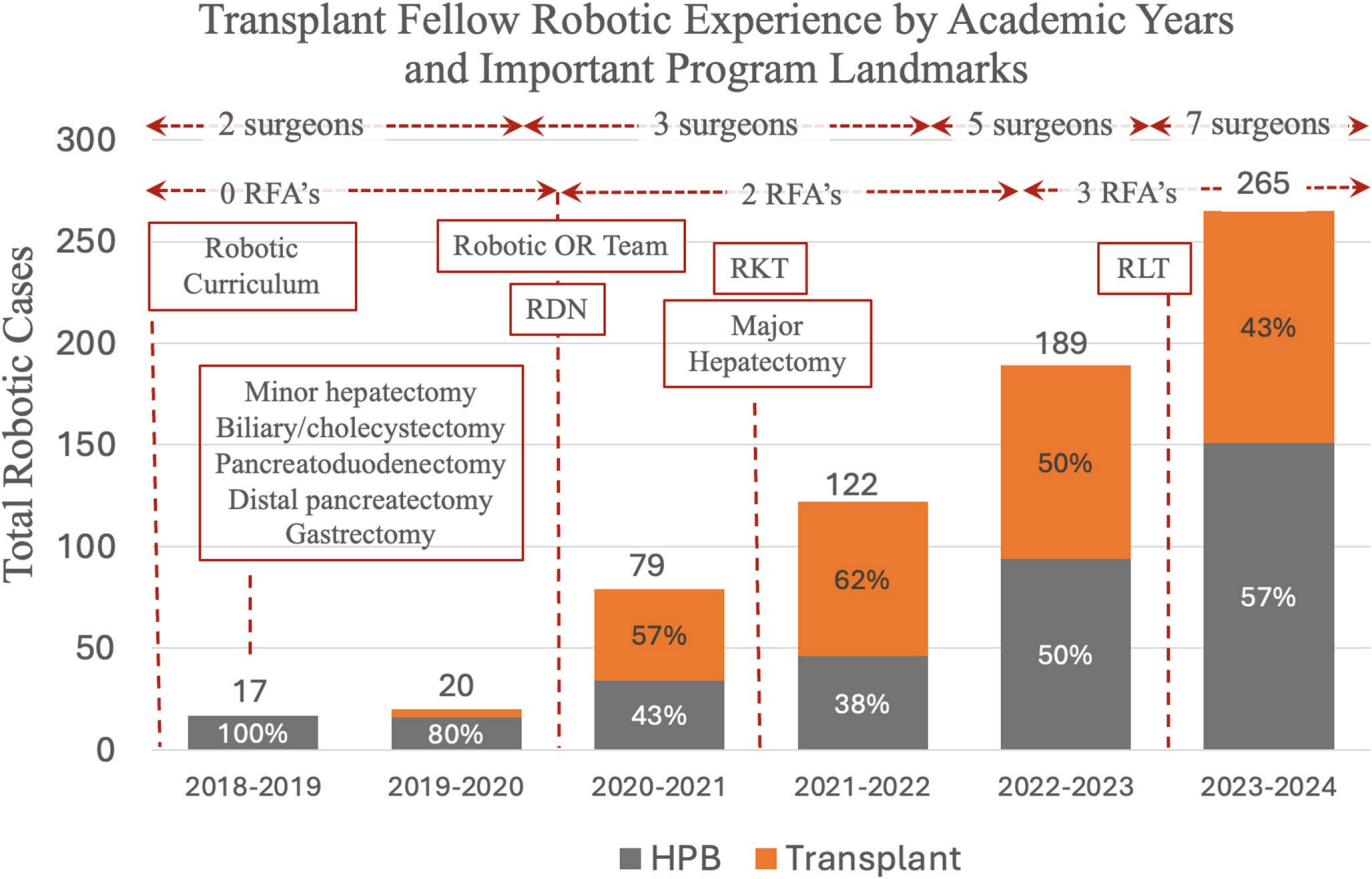
Bar graph demonstrating robotic fellow experience during the study period by academic year. Important landmarks have been included to give an idea about timeline of overall robotic program growth relative to fellow training. (RFA = robotic first assist, RDN = Robotic donor nephrectomy, RKT = Robotic kidney transplant, RLT = Robotic liver transplant)

Figure 2 illustrates the number of cases with fellow participation for each academic year of the study period. This figure again demonstrates an increase in the number of fellow-performed cases for each successive year of the study for both transplant and HPB. Landmark events in the development of the robotic program have been marked in the figure to contextualize their impact on fellow robotic case numbers.

The details of the 10-question survey administered to the 9 ASTS fellows are included as Online Resource 3. Fellow response to the survey was 100%. Eight of the 9 fellows had some robotic experience prior to starting fellowship, with residency robotic case volumes ranging between 30 and 100 cases. The majority (> 50%) of the residency robotic experience was in the role of bedside assistant. The fellows reported a varying level of confidence in their ability to independently perform index transplant and HPB operations at the end of fellowship training (Online Resource 4). Eight of the 9 fellows (89%) felt they could independently perform robotic living donor nephrectomy and minor hepatectomies, seven (78%) felt comfortable performing distal pancreatectomy and splenectomy, and four (44%) reported high comfort level with robotic kidney transplantation and major hepatectomy. All participating fellows felt that robotic experience during fellowship is important to a career in transplant surgery in present times. Seven of the 9 (78%) fellows believed that the indications for robotics in transplant will continue to broaden significantly, with 2 of the most recent fellows indicating a belief that the robotic approach would become the gold standard for many routine transplant and HPB operations. Online Resource 5 details the current practices of the 9 fellows, including the common robotic operations they perform. All graduated fellows have successfully incorporated robotic surgery in their practice.

## Discussion

To our knowledge, this is the first study to systematically examine the integration of robotic training during a transplant surgery fellowship. Our seven-year experience demonstrates that a structured, curriculum-based approach can be successfully implemented to equip transplant fellows with a valuable and increasingly essential skill set. This foundation prepares them to meet the evolving demands of abdominal transplant and hepatobiliary surgery, where minimally invasive approaches—particularly robotics—are gaining traction [5, 6, 17–19].

The insights gained from our trainee survey mirror national trends among HPB and transplant fellows, confirming growing enthusiasm and demand for robotic training [10]. All nine fellows in our program reported satisfaction with their robotic experience, and notably, all of them have gone on to incorporate robotic surgery into their clinical practices. We believe that this highlights the effectiveness of a well-designed, progressive curriculum tailored to a high-volume, high-complexity center.

That said, several limitations should be acknowledged. First, there is significant variability across transplant programs in terms of case mix and surgical scope which makes standardization of robotic training challenging [14, 15, 19]. Our institution is somewhat unique in offering a diverse mix of transplant and HPB operations, including general surgery and “bread-and-butter” HPB cases. These operations serve as an ideal platform for building core robotic skills before progressing to more advanced maneuvers required in living donor and transplant procedures [8]. Not having access to these kinds of ‘starter’ cases can add to the challenge of training fellows in more complex cases. Another limitation with the paper is related to the considerable variation in robotic expertise among transplant surgeons nationally, which can often impact how much autonomy fellows are granted in the OR [10, 13]. Most of the transplant faculty at our institution are experienced robotic surgeons and their approach to fellow training might be different from that in other programs.

We believe that our study provides a preview of the natural evolution of robotic training in transplant programs across the country—both in curriculum structure and fellow participation. The complexity and numbers of cases performed at our institution increased with faculty experience. We also noted an improvement in the robotic skills of the incoming fellows due to their increasing exposure to robotic surgery during residency. Not surprisingly, it appears from our experience that this combination of increasing faculty experience and robotic skills of incoming fellows considerably enhanced the fellowship training experience in both case volume and complexity. We can probably expect to see the same trend nationally in the next few years- as experience among both faculty and trainees continues to grow, the overall quality of robotic training in transplant fellowships is likely to improve.

Despite institutional differences, we believe there are several core principles from our program that are broadly applicable and can be adapted to other settings, regardless of faculty expertise or procedural scope:

- *Institution-specific robotic curriculum*: Fellowship programs should develop robotic curricula that reflects their specific case mix and faculty capabilities. Our early curriculum emphasized basic robotic skills and system troubleshooting given the minimal robotic experience of incoming fellows. Over time, as the incoming fellows demonstrated increasing proficiency with the basic concepts of robotics due to the exposure during residency, the training focused more on advanced dissection techniques, vascular anastomoses, and operation-specific pathways [17–20].
- *Use of training models*: Simulation-based training significantly accelerates robotic skill acquisition. Bio-tissue models offer high haptic fidelity and are effective in teaching skills transferable to complex operations like pancreaticoduodenectomy and biliary reconstruction [16, 20, 21]. At our center, the robotic kidney transplant (RKT) model has proven particularly effective for teaching microvascular anastomoses using 6 − 0 or 7 − 0 Prolene. We have also employed cadaveric and pig labs for advanced dissection training. Perfused cadavers, while costly, provide unparalleled realism for simulating living donor and transplant procedures and have been especially helpful in preparing both fellows and junior faculty [22].
- *Operation-specific training pathways*: Structured intraoperative pathways can provide fellows with a graduated path to autonomy. For example, we previously validated a performance-based training pathway for robotic donor nephrectomy using both subjective (MO-SCORE) [11] and objective metrics (fellow console time, handoffs) [12]. These metrics showed that fellows achieved proficiency after 14–18 cases and independence after 32–33 cases [12]. Our ongoing validation of the ARKTIC model for RKT shows promising results, with fellows achieving complete independence (100% console time) after structured training. These pathways are reproducible and offer opportunities for cross-institutional collaboration [11, 12, 15].
- *Creating console opportunities*: Robotic skill development is cumulative, and every opportunity at the console matters. Dual-console systems facilitate handoffs and minimize disruption. This creates more opportunities for fellows to take control and easier for attendings to teach during more complex portions of the case [12, 23]. Incorporating a dedicated robotic first assistant (RFA) also enhances fellow participation [13]. After integrating RFAs into our program, we observed a jump in fellow participation in robotic cases from 59% to over 90%, and their console involvement increased fivefold—from 19% to 94% [13].

We are still some time away from reaching a point where robotic training in transplant surgery is consistent across all fellowship programs or where a single, standardized curriculum fits everyone. But in the meantime, there are practical and collaborative ways we can move the field forward in addition to the methods outlined earlier. National hands-on courses, where experts teach robotic transplant operations in a structured setting, could help lay the groundwork for shared surgical standards. Fellowship exchange opportunities could also allow trainees to explore robotic techniques not available at their home institutions. Additionally, collaboration between transplant/HPB surgical societies and fellowship committees can facilitate standardization of robotic training in a manner that is safe, structured, and adaptable to the diverse needs of programs and trainees.

**In summary**, our experience demonstrates that a structured, curriculum-driven approach to robotic training can be successfully integrated into transplant surgery fellowships. Despite institutional differences, core strategies—such as tailored curricula, simulation-based skill development, operation-specific training pathways, and intentional creation of console opportunities—can be adapted to diverse settings. As robotic surgery continues to expand within transplant and HPB surgery, training programs must evolve accordingly to prepare the next generation of surgeons with the skills needed to meet future challenges.

## Supplementary Information

Below is the link to the electronic supplementary material.

Supplementary Material 1

Supplementary Material 2

Supplementary Material 3

Supplementary Material 4

Supplementary Material 5

## Data Availability

The data that support the findings of this study are available from the corresponding author upon reasonable request.

## Abbreviations

AHPBA: American Hepato-Pancreato-Biliary Association
ASTS: American Society of Transplant Surgeons
HPB: Hepato-Pancreato-Biliary
KT: Kidney Transplant
LT: Liver Transplant
RDN: Robotic Donor Nephrectomy
RFA: Robotic First Assist
RKT: Robotic Kidney Transplant
RLT: Robotic Liver Transplant
RNFA: Registered Nurse First Assist
RFA: Robotic First Assist
