# Supplementary figures and images for: Robotic training in transplant surgery fellowship: shaping the next generation of transplant surgeons
Source: J Robot Surg. 2026 Feb 19;20(1):268. doi: 10.1007/s11701-026-03215-w (PMC12920301; doi:10.1007/s11701-026-03215-w)

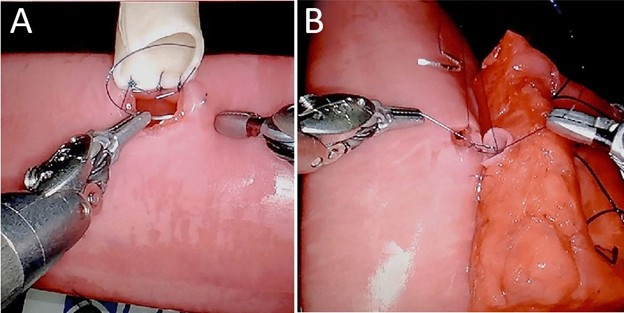

Supplement: Supplementary file 1 — Supplementary Material 1 [file 11701_2026_3215_MOESM1_ESM.jpg]

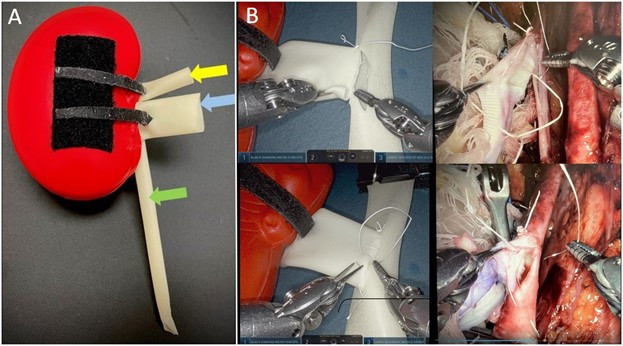

Supplement: Supplementary file 2 — Supplementary Material 2 [file 11701_2026_3215_MOESM2_ESM.jpg]

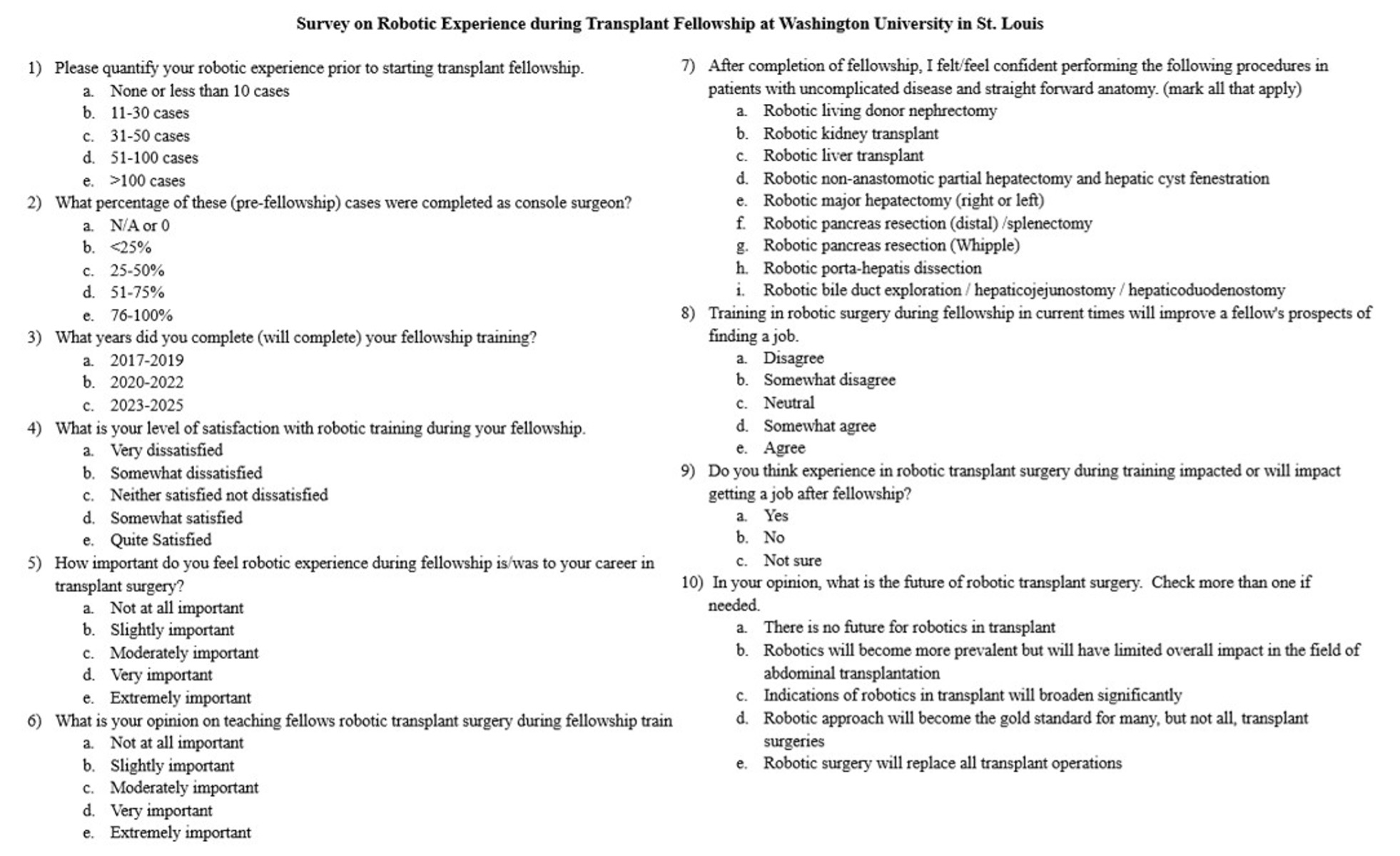

Supplement: Supplementary file 3 — Supplementary Material 3 [file 11701_2026_3215_MOESM3_ESM.jpg]

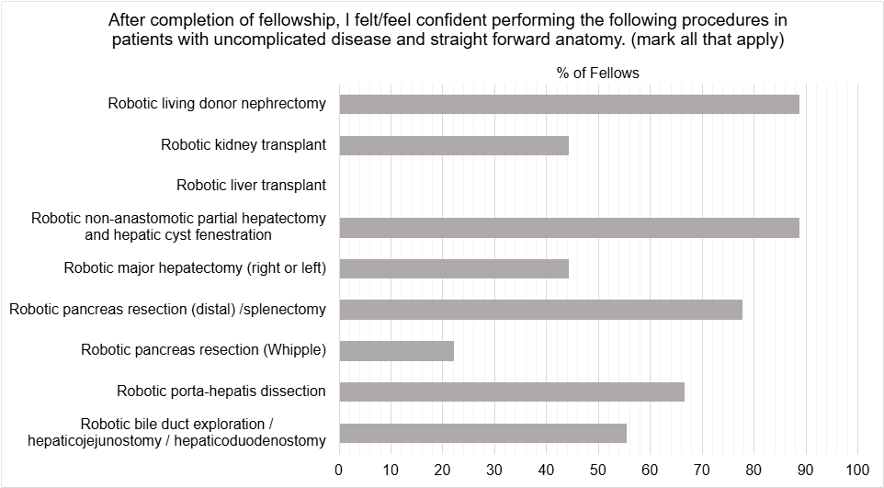

Supplement: Supplementary file 4 — Supplementary Material 4 [file 11701_2026_3215_MOESM4_ESM.png]
