# Supplementary material for: Robotic training in transplant surgery fellowship: shaping the next generation of transplant surgeons
Source: J Robot Surg. 2026 Feb 19;20(1):268. doi: 10.1007/s11701-026-03215-w (PMC12920301; doi:10.1007/s11701-026-03215-w)
Supplement: Supplementary file 5 — Supplementary Material 5 [file 11701_2026_3215_MOESM5_ESM.pdf]

Online Resource 5: Current scope of practice of the 9- fellows and details of the type of robotic cases they are performing in practice. Fellows are listed in chronological order and date of graduation included in parenthesis.

	Scope of Current Practice	Robotic Cases Currently Performed
Fellow 1<br>(2019)	Liver transplant and HPB	Minor and major hepatectomy<br>Biliary
Fellow 2<br>(2020)	Multivisceral transplant and HPB	Living donor nephrectomy<br>Hepatic resections<br>Biliary
Fellow 3<br>(2021)	Liver transplant and HPB	Living donor hepatectomy<br>Hepatectomy<br>Biliary
Fellow 4<br>(2022)	Kidney transplant	Living donor nephrectomy
Fellow 5<br>(2023)	Kidney transplant	Living donor nephrectomy
Fellow 6<br>(2023)	Multivisceral transplant and HPB	Living donor nephrectomy<br>Kidney transplant<br>Major and minor hepatectomy<br>Pancreatectomy<br>Biliary<br>Gastric
Fellow 7<br>(2024)	Multivisceral transplant<br>HPB	Robotic donor nephrectomy<br>Kidney transplantation<br>Hepatic resection
Fellow 8*<br>(2025)	Still in training with plans to incorporate robotic transplant and HPB in practice.	
Fellow 9*<br>(2025)	Still in training with plans to incorporate robotic transplant in practice.	
